# Near-infrared spectroscopy analysis to predict urinary allantoin in dairy cows

**DOI:** 10.3168/jdsc.2024-0641

**Published:** 2024-12-16

**Authors:** Leonardo A.C. Ribeiro, Guilherme L. Menezes, Tiago Bresolin, Sebastian I. Arriola Apelo, Joao R.R. Dórea

**Affiliations:** 1 Department of Animal and Dairy Sciences, University of Wisconsin–Madison, Madison, WI 53706; 2 Department of Animal Sciences, University of Illinois Urbana-Champaign, Urbana, IL 61801; 3 Department of Biological Systems Engineering, University of Wisconsin–Madison, Madison, WI 53706

## Abstract

Accurate quantification of rumen microbial proteins is essential in dairy cow nutrition to estimate the ruminal escape of dietary protein and microbial yield. Current quantification methods rely on indirect measurements using purine derivatives (PD). However, these methods require specialized laboratory equipment and trained personnel, resources which are often not available in farm settings. Near-infrared spectroscopy (NIR) has emerged as a powerful tool for predicting the attributes of biological samples, including meat, corn, soybeans, and liquids. Given that allantoin is the primary component in PD, this study aims to (1) develop a predictive model for allantoin levels in urine using NIR and (2) identify key spectral regions for future applications. A total of 182 urine samples were collected from 182 Holstein cows for colorimetric analysis of allantoin and spectral analysis. The raw spectra were preprocessed using scatter correlation methods and spectral derivatives. The partial least squares regression model achieved an R^2^ of 0.55, a concordance correlation coefficient of 0.73, and a root mean squared error of prediction (RMSEP) of 3.63 mmol/L to predict allantoin concentration from the spectra data set without preprocessing. However, the use of the first derivative (FirstDev) as a preprocessing step reduced the RMSEP from 3.63 mmol/L to 3.25 mmol/L and increased the R^2^ from 0.55 to 0.62. The FirstDev improves spectral resolution by eliminating the constant baseline, potentially explaining the improved model accuracy. Our method has the potential to evaluate the passage rate of microbial protein represented by the changes in urinary allantoin extraction and the potential to be used for AA dietary balance, thereby improving environmental sustainability and profitability in dairy farms.

## Highlights

• Spectral preprocessing techniques increase the R^2^ and RMSEP.
• Spectral analysis predicts allantoin with R^2^ of 0.62 and RMSEP of 3.25 mmol/L.
• Urine spectra evaluate microbial protein passage via urinary allantoin.

## Graphical Abstract

**Figure:**
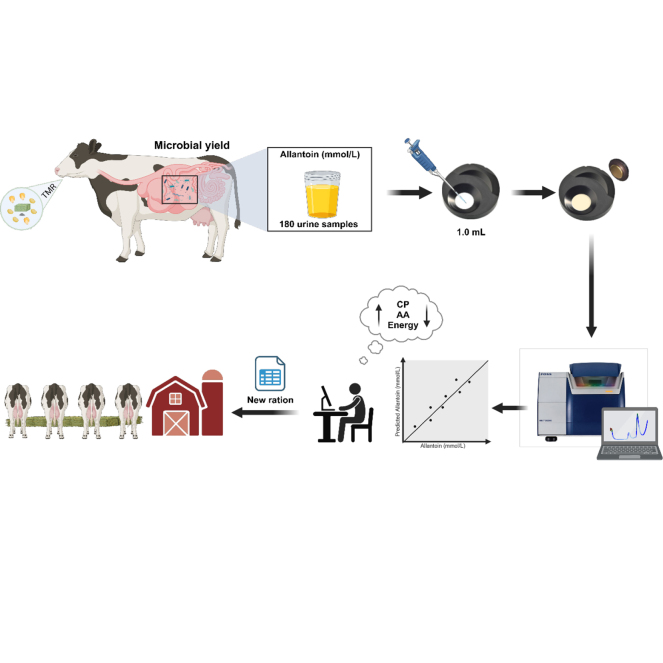
**Summary:** This study developed a partial least squares regression (PLSR) model for measuring allantoin levels in dairy cow urine using near-infrared spectroscopy. The PLSR achieved a coefficient of determination (R^2^) of 0.55, a concordance correlation coefficient of 0.73, and a root mean squared error of prediction (RMSEP) of 3.63 mmol/L. With first derivative preprocessing, the RMSEP improved to 3.25 mmol/L, and the R^2^ increased to 0.62. This model can evaluate microbial protein passage via urinary allantoin extraction, which may improve the dietary balance of amino acids (AA), enhancing farm profitability and environmental sustainability.

Rumen microbes have great metabolic flexibility to use different N sources for protein synthesis, including non-amino N sources such as urea from dietary origin or recycled from circulation through saliva or the rumen epithelium. This makes rumen microbial protein among the cheapest metabolizable nutrients for a lactating cow (Binggeli et al., 2021), and still, it is highly digestible and has an excellent AA profile in relation to milk proteins (Sok et al., 2017). Therefore, maximizing microbial protein yield could help to reduce the use of expensive bypass proteins, potentially increasing income over feed cost, milk components yield, and N efficiency (Haque et al., 2012), as well as reducing environmental nitrogen emissions, including ammonia, nitrite, and nitrous oxide (Hristov et al., 2019). Despite its critical role in ruminant nutrition, we lack a reliable, rapid, and noninvasive method to quantify rumen microbial protein yield and flow to the small intestine.

In this context, several researchers have proposed an indirect measurement, the ratio of purine derivatives (**PD**) to creatinine, as an indicator of microbial synthesis (Timmermans et al., 2000; Tebot et al., 2002; Schager et al., 2003; Del Valle et al., 2023). Purine bases are integral components of the nucleic acids found in microbial cells. The nucleic acids synthesized by rumen microorganisms undergo enzymatic degradation into purine and pyrimidine derivatives, such as uric acid and allantoin. These final products are eliminated in urine, with allantoin constituting the largest proportion (Tebot et al., 2002). The use of urinary allantoin as an indirect method for estimating microbial proteins has proven to be a valuable research tool in assessing microbial protein yield (Tebot et al., 2002). Broderick and Merchen (1992) investigated the relationship between total purine excretion (encompassing allantoin, uric acid, xanthine, and hypoxanthine) and abomasal purine infusion. They observed a linear relationship between them, suggesting that urinary purine excretion can be employed as a noninvasive method for estimating microbial protein synthesis. Therefore, higher allantoin concentrations indicate increased microbial protein production, a high-quality and cost-effective protein source for cows (Johnson et al., 1998).

One limitation of applying PD ratios as a routine practice in dairy farms for nutritional adjustments is that the current method for evaluating allantoin proposed by Chen and Gomes (1992) is based on colorimetric evaluation (de Kássia Gomes et al., 2024). This process requires specialized laboratory equipment and trained personnel, resources that may not be available in a farm setting. As an alternative, near-infrared spectroscopy (**NIR**) has emerged as a powerful and widely used tool for predicting the attributes of biological samples, including meat, corn, and soybeans (Büning-Pfaue, 2003). Compared with other techniques, NIR exhibits greater sensitivity to molecular interactions and chemical groups (Shi et al., 2019), which can be useful for predicting allantoin concentrations in urine.

However, evaluating liquid samples can pose significant challenges, particularly due to the strong absorption of water in the NIR region (Büning-Pfaue, 2003). This absorption tends to dominate the spectral curve in samples that contain more than 80% water, such as milk, urine, and plasma. As a result, the spectral signal closely resembles that of water, which can reduce the effectiveness of models in extracting important signals from the spectra for phenotyping (Büning-Pfaue, 2003). Despite the prevalent influence of water, previous studies have reported that NIR spectroscopy can detect subtle changes, such as variations in the amount of creatinine (Kinoshita et al., 2016). Therefore, these findings underscore the importance of exploring spectral urine analyses to estimate PD. Additionally, to our knowledge, predicting allantoin concentration using NIR from urine samples is a novel and unexplored approach. In this context, this study aims to (1) develop a predictive model for allantoin levels in urine and (2) identify key spectral regions for future applications.

All experimental procedures in this study received ethical approval from the Institutional Animal Care and Use Committee at the University of Wisconsin–Madison (Madison, WI). A total of 182 urine samples were collected from 182 Holstein cows, with an average milk production of 41 ± 12 L/d and 164 ± 112 DIM, at the University of Wisconsin–Madison, Emmons Blaine Dairy Cattle Research Center (Arlington, WI). The number of cows in their first, second, third, and subsequent lactations were 32, 54, 50, and 46, respectively. The cows were fed a TMR once daily at 0700 h, and the feed bunk was adjusted as needed to fulfill the nutritional needs. The urine samples were collected in the morning at 0600 h and stored in clean plastic disposable urine sample containers. An aliquot of 15 mL of urine was then diluted with 60 mL of 0.072 *N* H_2_SO_4_ to maintain a pH below 4 and minimize ammonia loss and stored at −20°C until analysis.

The allantoin quantification was performed in triplicate using the colorimetric method described by Young and Conway (1942), adapted for a 96-well plate reader (Ashour et al., 1987). The ground truth value of allantoin used to train the models was assumed to be the average of the triplicate measurements. For NIR analysis, 1,000 µL of urine from the same sample used for nonesterified fatty acid quantification was dispensed into a slurry cup equipped with a 0.5-mm gold reflector. All samples were subjected to analysis using FOSS DS2500 spectrometers (Foss, Hillerød, Denmark), resulting in the generation of a single spectrum for each urine sample. The Foss NIR spectrum comprises 4,200 data points, which reflect the absorption of infrared light across a wavelength range of 400 to 2,500 nm within the urine sample. The spectra were acquired by transmitting incident light through the sample, reflecting it off the gold reflector, and then passing the reflected light back through the sample. Although the samples were collected using a benchtop NIR, we selected the 400 to 1,050 nm range (strategy 2) from the raw spectrum for future applications, aiming to simulate the performance of portable NIR devices that usually cover the same spectral range (Crocombe, 2018).

The raw spectral data (one spectral signal per urine sample) were preprocessed using scatter correction techniques and spectral derivative methods. Multiplicative scatter correction (**MSC**) and standard normal variate (**SNV**) were used for scatter correlation analysis. The first derivative (**FirstDev**) and second derivative were used as spectral derivatives. These transformations were selected based on their common usage in literature (Bresolin and Dórea, 2020). All transformations were carried out using the package pyspectra using the Python language. All datasets, including the raw data and data from the preprocessing method, were used for predictions, and the preprocessing method that yielded the lowest root mean squared error of prediction (**RMSEP**) was used to identify key spectral regions for future applications (Figure 1).

**Figure 1:**
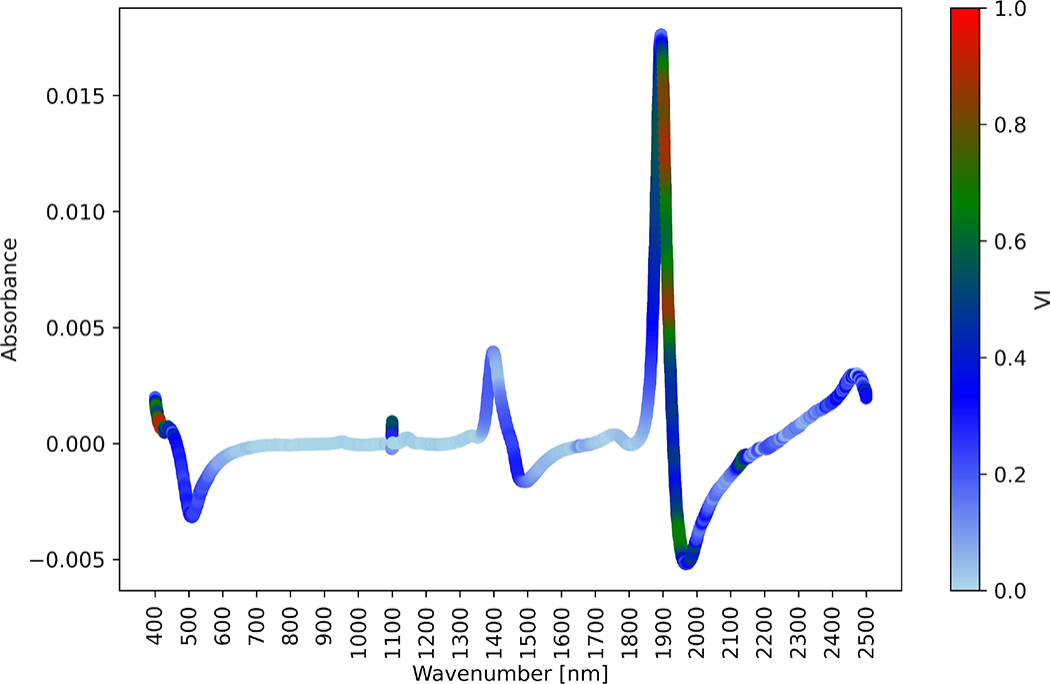
Variable importance (VI) to predict allantoin concentration. Absorbance and wavenumbers (nm) were obtained from urine samples using near-infrared spectroscopy through leave-one-out cross-validation (n = 182). The VI is represented on a color scale ranging from light (blue, indicating lower importance) to dark (red, indicating higher importance). The graph illustrates the VI based on the FirstDev + MSC approach.

A leave-one-out cross-validation approach was employed to evaluate the model's predictive performance. In this approach, data from a specific cow was used as a validation set, while data from the remaining cows were used to train the partial least squares regression (**PLSR**). The number of latent variables was determined through a 10-fold cross-validation using the training dataset, and the number of latent variables was selected based on the lowest RMSEP. During this process, a maximum of 40 latent variables was set. To assess the model predictive ability, RMSEP, R^2^, and the concordance correlation coefficient (**CCC**; Liao, 2003) were calculated, as shown in Table 1 and Figure 2. All predictive analyses were performed using the statistical software R (version 4.2.1; R Core Team, 2019).

**Table 1:** Relationships between predicted and observed allantoin values

Variable^1^	Observed	Predicted value						
		Raw	FirstDev	SecondDev	SNV	MSC	FirstDev + MSC	FirstDev + SNV
Strategy 1^2^ (400 to 2,500)								
Mean (mmol/L)	18.5	18.5	18.5	18.5	18.6	18.5	18.5	18.5
R^2^		0.55	0.62	0.40	0.53	0.54	0.62	0.62
CCC		0.73	0.78	0.60	0.72	0.73	0.78	0.78
RMSEP		3.63	3.27	4.13	3.7	3.65	3.25	3.26
RMSEP (%)		19.6	17.6	22.3	20.0	19.7	17.6	17.6
Strategy 2^3^ (400 to 1,050 nm)								
Mean (mmol/L)	18.5	18.5	18.5	18.6	18.5	18.5	18.5	18.5
R^2^		0.56	0.54	0.30	0.52	0.52	0.52	0.52
CCC		0.73	0.71	0.53	0.69	0.69	0.69	0.69
RMSEP		3.51	3.58	4.58	3.69	3.69	3.69	3.69
RMSEP (%)		19.0	19.4	24.8	19.9	19.9	19.9	19.9

1 CCC = concordance correlation coefficient; RMSEP = root squares mean prediction error of prediction; RMSEP (%) = root squares mean prediction error of prediction divided by the mean observed value.

2 The predominant latent variables in strategy 1 using raw spectral data, FirstDev, second derivative (SecondDev), SNV, MSC, FirstDev + MSC, and FirstDev + SNV, were 9, 5, 3, 7, 7, 4, and 4, respectively.

3 The models trained using strategy 2 with raw spectral data, FirstDev, SecondDev, SNV, MSC, FirstDev + MSC, and FirstDev + SNV, had predominant latent variables of 4, 1, 12, 2, 2, 2, and 2, respectively.

**Figure 2:**
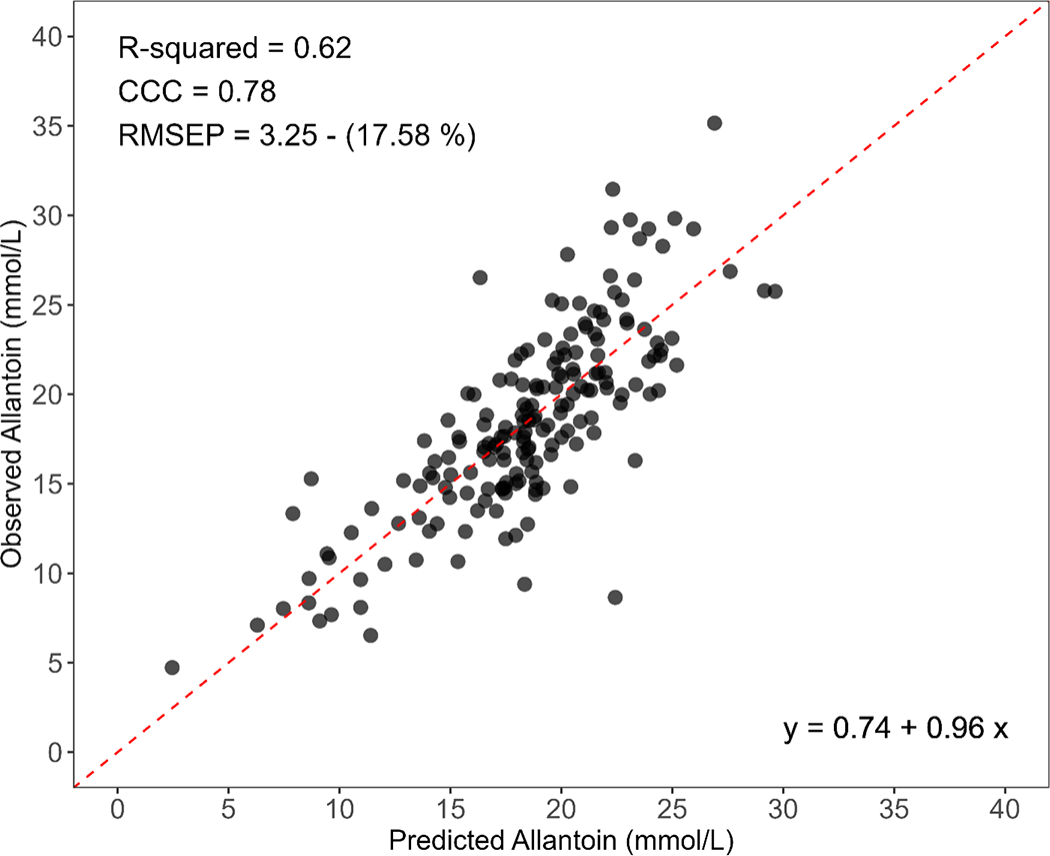
Relationship between predicted allantoin concentration (mmol/L) based on near-infrared spectra data and observed allantoin concentration in dairy cow urine (n = 182). The raw spectral data were preprocessed using FirstDev + MSC. The most frequent model had 4 latent variables. RMSEP = root mean square error of prediction; CCC = concordance coefficient of correlation.

The measurement of rumen microbial yield using allantoin is well-established and favored due to its simplicity and noninvasive nature (Hristov et al., 2019; Zacaroni et al., 2022; Oliveira et al., 2023). In our study, NIR spectroscopy was employed to analyze urine samples. The model trained with the raw spectrum achieved an R^2^ of 0.55, a CCC of 0.73, and an RMSEP of 3.63 mmol/L using a PLSR model to predict allantoin (Table 1). Preprocessing the samples with the FirstDev improved the accuracy by 10%, reducing the RMSEP from 3.63 mmol/L to 3.27 mmol/L and increasing the R^2^ from 0.55 to 0.62 (Table 1; Figure 2). The FirstDev improves spectral resolution by eliminating the constant baseline, potentially explaining the improved model accuracy. Although Bresolin and Dórea (2020) reported in their review paper some successful cases using the first derivative combined with MSC and SNV, we observed only minor improvements in our study (Table 1).

Even though the data were collected using a benchtop NIR instrument under controlled conditions, restricting the spectral range to 1,050 nm yielded promising outcomes with the raw spectra (Table 1). Although the application of FirstDev preprocessing improved the accuracy of the model using the full spectral range, these techniques in strategy 2 resulted in lower accuracy and precision. Therefore, NIR with a lower spectral range may not be as accurate compared with benchtop NIR. However, the trade-off between cost, ease of use, and precision will determine the most suitable instrument for each scenario. Considering that the spectral range used in strategy 2 is the same as in regular portable NIR (Crocombe, 2018), these sensors should be evaluated in future studies.

This study focused on predicting allantoin levels to estimate PD. Allantoin account for over 76.5% of total PD (Johnson et al., 1998) and can exhibit concentrations 7 times higher than those of uric acid (González-Ronquillo et al., 2004; Totty et al., 2013; Ferreira et al., 2023). However, both allantoin and uric acid can be crucial for estimating ruminal microbial yield and nitrogen flow. Johnson et al. (1998) noted a strong linear relationship between nitrogen flow and both allantoin and uric acid, with R^2^ values of 0.68 and 0.65, respectively. Furthermore, they demonstrated that uric acid is approximately twice as sensitive compared with allantoin in reflecting small changes in microbial nitrogen production. Therefore, future research should also evaluate uric acid levels to improve the overall accuracy of PD estimation, thereby improving assessments of ruminal microbial yield.

One next step to accurately determine microbial yield in the rumen is to evaluate the total urine production (Dórea et al., 2017). This measure can be estimated using creatinine concentration as described by Chen et al. (2004). Previous research (Shaw et al., 1996; Pezzaniti et al., 2001; Kinoshita et al., 2016) has shown encouraging results in predicting creatinine levels in urine samples using NIR. Suzuki et al. (2020) demonstrated promising results in using NIR to predict the Na-to-creatinine ratio in urine samples, offering a method to measure 24-h urinary sodium excretion. Therefore, future research also should concentrate on predicting creatinine levels to estimate urine production and determine microbial yield in the rumen.

Spectral analysis can be important for estimating PD as a metric for microbial yield and can also predict complex traits such as DMI and digestible DMI (**dDMI**). These traits are crucial for optimizing feed nutrition to enhance animal performance and feed efficiency (Brown et al., 2022). Estimating DMI on farms is challenging and typically relies on equations using animal characteristics such as BW, milk yield, and DIM (Brown et al., 1977), as well as dietary components such as ADF, NDF, and diet degradability (Allen et al., 2019). However, these equations do not account for how animals interact with their diet, such as the increase in digestible organic matter and the resulting improvement in ruminal microbial yield.

In this context, Dórea et al. (2017) proposed a model to predict DMI and dDMI based on PD, specifically allantoin and uric acid. They found a strong relationship between DMI and the PD-to-creatinine ratio index (**PDCindex**; the sum of allantoin and uric acid, divided by urinary creatinine and multiplied by metabolic BW), with R^2^ values of 0.90 and 0.96 for dairy and beef cattle, respectively. A strong relationship was also observed between dDMI and the PDCindex, with R^2^ values of 0.91 and 0.97 for dairy and beef cattle, respectively. Although both DMI and dDMI showed strong relationships with PD, using the full spectral signal can be more informative and may improve the accuracy of predictions.

Estimating DMI and ruminal microbial protein yield can clarify how different diets affect ruminal metabolism. Many dietary factors can reduce microbial protein synthesis, with energy and protein being the most influential (Clark et al., 1992). Understanding these factors is a priority in dairy cow nutrition, as the rumen microbiome can contribute 60% to 90% of the metabolizable protein (Huws et al., 2018). Consequently, diets are formulated to supply AA in the small intestine, complementing microbial protein synthesized in the rumen and endogenous protein (Clark et al., 1992). However, measuring the pool of AA under farm conditions is challenging and is currently assessed using predictive equations (Lapierre et al., 2023). Therefore, measuring the amount of allantoin excreted in urine using NIR can be a reliable method to estimate ruminal microbial growth and metabolizable AA supply to the cow, with important implications for AA metabolism and improved decision-making to reduce dietary CP and N excretion.

Such information is important because protein is typically the most expensive component in animal diets, and overfeeding it can lead to economic and environmental consequences (Sheehy et al., 2020). Recent studies have explored the strategy of AA supplementation (Higgs et al., 2023; Seleem et al., 2024) to reduce the overall CP content in diets, potentially improving milk yield and feed efficiency (Irawan et al., 2023). However, despite supplementation, when CP is reduced in diets, an overestimation of AA can occur (Van den Bossche et al., 2023), limiting milk production and feed efficiency. Normally, diets that include bypass proteins or rumen-protected AA are more expensive per kilogram of dry matter (Elsaadawy et al., 2022). In this context, a lack of productive response can result in lower profitability on dairy farms. Therefore, NIR as a tool to evaluate ruminal microbial yield may support nutritionists in making better-informed decisions regarding a decrease in CP in diets without decreasing animal performance.

Leave-one-out cross-validation is a widely used strategy, especially when the sample size is limited, to evaluate the predictive performance of models. In this particular trial, although it would be ideal to test the model using an external data set for further validation, cross-validation techniques such as leave-one-out and k-fold serve as effective alternatives. These methods provide a more reliable assessment than simply fitting the model to the training data, which often leads to overoptimistic results due to overfitting. Cross-validation mitigates this risk by ensuring that the model's performance is tested on different subsets of data. Bresolin and Dórea (2020) have discussed cross-validation strategies in detail using spectral data in livestock systems, highlighting their importance in such applications as well as the advantages and disadvantages of cross-validation strategies. Furthermore, Dórea et al. (2017) demonstrated the importance of using external validation sets when available, showing that external validation offers a more accurate measure of a model's generalizability.

In conclusion, NIR showed promising results for predicting urine allantoin. Future studies should explore this approach across different farms and dietary conditions. Predicting allantoin from urine samples can provide valuable information to support well-informed decisions about balancing essential amino acids and potentially reducing crude protein in diets without compromising performance. This could enhance environmental sustainability and profitability on dairy farms.
